# Nonfunctional ectopic adrenocortical carcinoma in the lung: A case report and literature review

**DOI:** 10.3389/fonc.2023.1100207

**Published:** 2023-02-16

**Authors:** Limin Nie, Shuyun Wang, Zongfeng Feng, Yuping Sun, Zhen Wang, Qi Dang, Aiqin Gao, Yajuan Lv

**Affiliations:** ^1^ Phase I Clinical Research Center, Shandong University Cancer Center, Jinan, Shandong, China; ^2^ Department of Oncology, Caoxian People’s Hospital, Heze, Shandong, China; ^3^ Department of General Surgery, Caoxian People’s Hospital, Heze, Shandong, China; ^4^ Department of Pathology, Caoxian People’s Hospital, Heze, Shandong, China; ^5^ Department of Radiation Oncology, Shandong University Cancer Center, Jinan, Shandong, China; ^6^ Department of Oncology, Shandong Key Laboratory of Rheumatic Disease and Translational Medicine, Shandong Lung Cancer Institute, the First Affiliated Hospital of Shandong First Medical University & Shandong Provincial Qianfoshan Hospital, Jinan, Shandong, China

**Keywords:** ectopic adrenocortical carcinoma, lung, nonfunctional, case report, adrenocortical carcinoma

## Abstract

**Background:**

Ectopic adrenocortical tissues and neoplasms are rare and usually found in the genitourinary system and abdominal cavity. The thorax is an extremely rare ectopic site. Here, we report the first case of nonfunctional ectopic adrenocortical carcinoma (ACC) in the lung.

**Case presentation:**

A 71-year-old Chinese man presented with vague left-sided chest pain and irritable cough for 1 month. Thoracic computed tomography revealed a heterogeneously enhancing 5.3 × 5.8 × 6.0-cm solitary mass in the left lung. Radiological findings suggested a benign tumor. The tumor was surgically excised upon detection. Histopathological examination using hematoxylin and eosin staining showed that the cytoplasm of the tumor cells was rich and eosinophilic. Immunohistochemical profiles (inhibin-a^+^, melan-A^+^, Syn^+^) indicated that the tumor had an adrenocortical origin. The patient showed no symptoms of hormonal hypersecretion. The final pathological diagnosis was non-functional ectopic ACC. The patient was disease-free for 22 months and is still under follow-up.

**Conclusions:**

Nonfunctional ectopic ACC in the lung is an extremely rare neoplasm that can be easily misdiagnosed as primary lung cancer or lung metastasis, both preoperatively and on postoperative pathological examination. This report may provide clues to clinicians and pathologists regarding the diagnosis and treatment of nonfunctional ectopic ACC.

## Introduction

Adrenocortical carcinoma (ACC) is a rare malignancy with an incidence of 1–2 cases/million persons/year and often has a poor prognosis (1). It has a predilection for the female gender (F:M; 1.5–2.5:1) and a bimodal age distribution with peaks in early childhood (under the age of 5 years) and middle-aged adulthood (40–50 years) (2, 3). Most cases of ACC are functional and usually exhibit endocrine symptomatology due to hormonal hypersecretion (4). Nonfunctional ACC is less common and lacks specific signs and symptoms (5, 6). Patients are identified incidentally or present with nonspecific complaints related to tumor overgrowth and mass effects, such as abdominal or back pain, or metastatic symptoms (5, 7–9). Nonfunctional ACC generally occurs in older adults and exhibits a rapidly worsening course (6). Ectopic ACC is a condition in which ACC appears in locations other than the adrenal glands, usually in the genitourinary system [ovary (10)] and abdominal cavity [abdominal wall (6), liver (4) and retroperitoneum (11, 12)] and occasionally in the nervous system (8). However, to the best of our knowledge, ectopic ACC has not been reported in the lungs. Here, we report an extremely rare case of nonfunctional ectopic ACC in the lung, detailing the clinical and pathological findings, treatment, and follow-up.

## Case report

## Clinical findings

**Figure 1:**
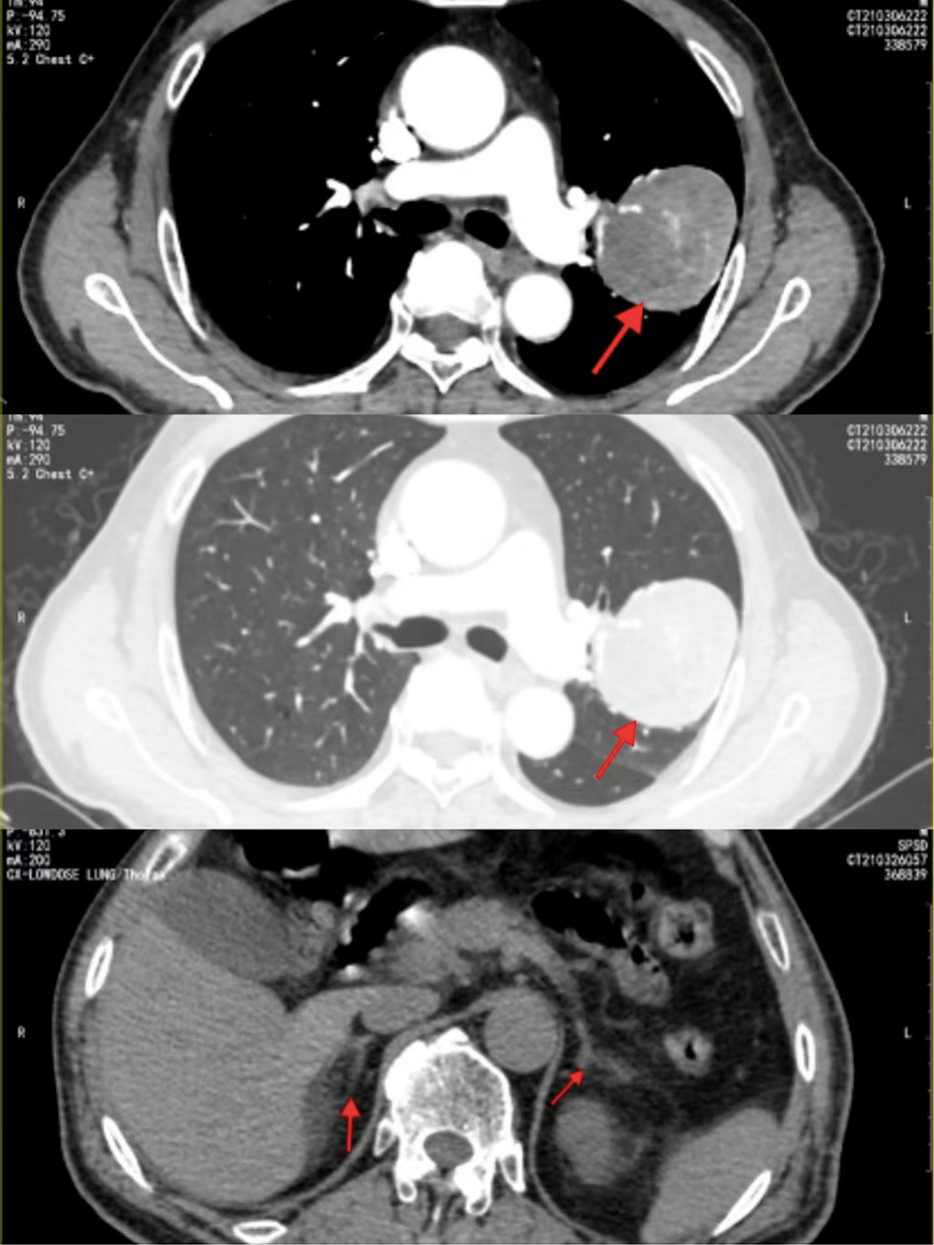
Contrast-enhanced CT revealed a heterogeneously enhancing solitary mass in the left lung;abdominal CT showed that the bilateral adrenal glands were normal in size and shape without abnormal mass.

A 71-year-old man with complaints of vague left-sided chest pain and irritable cough for 1 month was admitted to our hospital in March 2021. He had no chills, fever, hemoptysis, wheezing, or abdominal pain. He was healthy with no relevant medical or family history of diseases, such as hypertension or diabetes, and no history of smoking or alcohol consumption. The laboratory findings showed that only the prograstin-releasing peptide (ProGRP) level was elevated (198.7 pg/mL), other tests showed no abnormalities. Thoracic computed tomography (CT) revealed a 5.3 × 5.8 × 6.0-cm heterogeneously enhancing globose mass in the posterior segment of the left upper lung lobe with a smooth margin without obvious lobulation or burr (**Figure 1**), suggestive of a benign tumor. After adequate preparation, thoracoscopic surgery of the upper lobectomy of the left lung was performed.

Postoperatively, the pathological diagnosis was suspected to be of an extrapulmonary and adrenocortical origin. Abdominal CT showed that the bilateral adrenal glands and kidneys were normal in size and shape without abnormal mass (**Figure 1**). ^18^F-fluorodeoxyglucose positron emission tomography was recommended to assess the condition of the whole body for invisible lesions. However, the patient refused.

Repeated laboratory examination revealed that the ProGRP level reduced from 198.7 to 78.5 pg/mL without any other abnormalities. We performed the Cortisol, ACTH, DHEA-S and Aldosterone tests, They’re all in the normal range. As the patient had no relevant clinical symptoms of hormonal hypersecretion, estradiol or other hormone tests were not performed.

## Pathological findings

Gross inspection of the lobectomy specimen revealed a mass lesion measuring 16×9×4 cm. The surface of the mass was mostly smooth, covered by the visceral pleura, and ruptured through the smooth inner capsule in an area measuring 6.7×6.5×4 cm. Capsular tissues had prolapsed at multiple sites. Sectioning revealed well-circumscribed, multilocular, tan-brown to tan-yellow cut surfaces.

**Figure 2:**
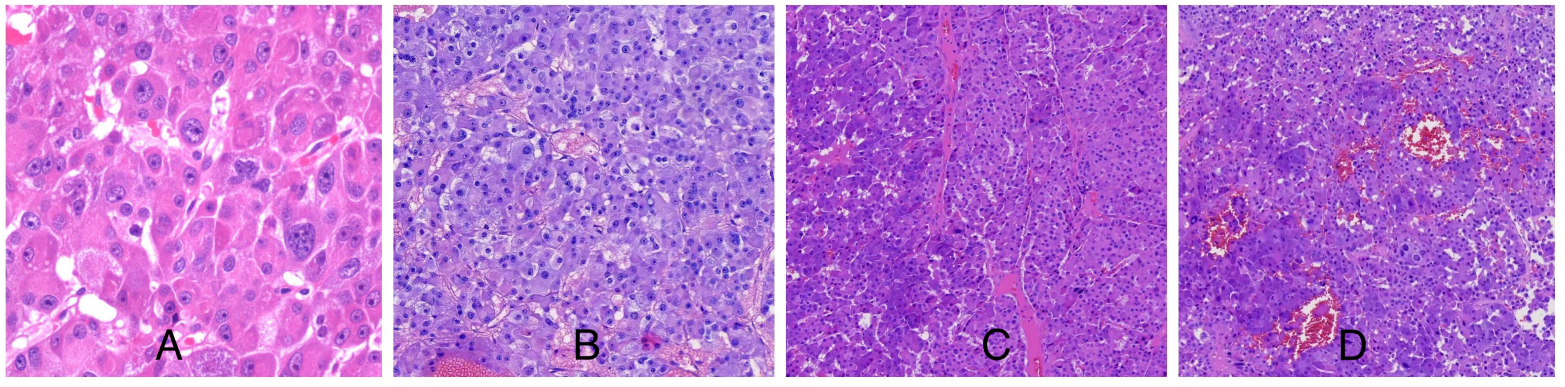
**(A)** The cytoplasm of the tumor cells was rich and eosinophilic and irregular karyotype; **(B)** Transparent bodies were seen in some tumor cells; **(C)** The tumor cells were pleomorphic and arranged in varying round blunt cell cords and cord-like structures; **(D)** Interstitial blood vessels were abundant in the tumor tissue.

**Figure 3:**
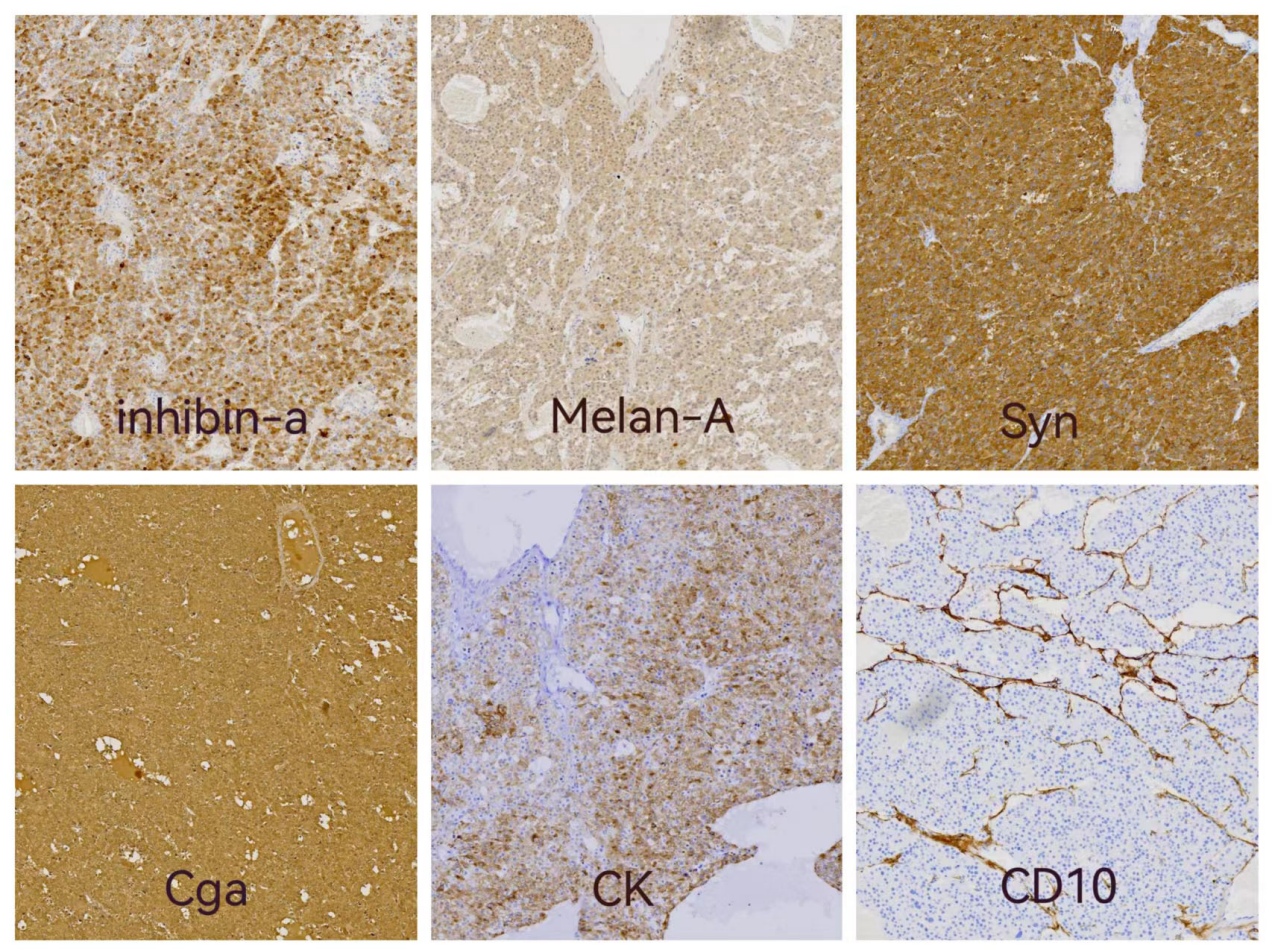
Immunohistochemistry results: immunostaining of inhibin-a, Melan A,Syn and CK showed positive in cytoplasmic. Cytoplasmic and nuclear staining were positive of Cga and CD10 showed positive in intercellular fibrous tissue.

Light microscopic examination of the hematoxylin and eosin-stained specimen revealed that the cytoplasm of the tumor cells was rich and eosinophilic. The tumor cells were pleomorphic and arranged in varying round blunt cell cords and cord-like structures, with transparent bodies in some cells. Interstitial blood vessels were abundant in the tumor tissue (**Figure 2**). The immunohistochemical results were: inhibin-α (+), melan A (+), Syn (+), CK (+), CgA (+), CD10 (+), TTF-1 (-), PAX8 (-), hepatocyte (-), and arginase-1 (-) (**Figure 3**). The Weiss score was 5, as the cells were pleomorphic and distributed in varying round blunt cell cords, with extensive necrosis, an irregular karyotype, and capsular invasion (13). After consultation with another pathological diagnostic center, combined with the clinical findings, morphology, immunohistochemistry findings, and Weiss score, primary pulmonary ectopic ACC was finally diagnosed.

## Treatment and follow-up

According to the staging criteria of ectopic ACC by the American Joint Committee on Cancer and European Network for the Study of Adrenal Tumors, the final pathologic stage was pT2N0M0, i.e., stage II.

Since ectopic ACC is a rare neoplasm, no consensus has been reached for treatment recommendations (14). After multidisciplinary consultation, mitotane was selected as the first-line treatment, however, mitotane is unavailable in China. Since ProGRP is still higher than normal after surgery, we choose chemotherapy as the adjuvant treatment (15). Therefore, chemotherapy with combined etoposide and cisplatin was used as the adjuvant treatment for 4 cycles 3 weeks postoperatively. The ProGRP level continued to decline to normal after two cycles of chemotherapy, and thoracic and abdominal CT showed no obvious abnormalities after two and four cycles of chemotherapy, respectively.

The patient was followed-up every 3 months after chemotherapy for 2 years, with the latest follow-up on August 25, 2022. The patient did not complain of discomfort, and physical examination, thoracic and abdominal CT, and laboratory examination showed no obvious abnormalities. The patient had a disease-free survival (DFS) of 22 months and is still under follow-up.

## Ethics statement

Ethics approval was obtained from Caoxian People’s Hospital. Data was collected following patient’s concent. Informed consent has been obtained from the patient for publication of the case report and accompanying images.

## Discussion

ACC is a rare malignancy with an incidence of 1–2 cases/million population and often has a poor prognosis (1), with a 5-year survival rate of 38%–27% (16, 17) and a 10-year survival rate of 7% (17). Approximately 40% cases of ACC are nonfunctional (3), with nonfunctional ectopic ACC being even rarer. Ectopic ACC is usually found in the genitourinary system and abdominal cavity (5, 6, 8–10, 12, 18–20) and occasionally found in the nervous system (8). However, to the best of our knowledge, ectopic ACC has not been reported in the lungs.

Here, we reported a rare case of nonfunctional ectopic ACC in the lung of a 71-year old man who presented with nonspecific complaints of vague left-sided chest pain and irritable cough caused by an overgrowth tumor. In the pathologic examination, both macroscopic and light microscopic findings were inconsistent with common lung tumors. Immunohistochemical results were: inhibin-a (+), melan-A (+), Syn (+), CK (+), CgA (+), TTF-1 (-), PAX8 (-), hepatocyte (-), and arginase-1 (-). Immunohistochemical profiles (inhibin-a^+^, Melan-A^+^, Syn^+^) indicated that the tumor had an adrenocortical but extrapulmonary origin. The Weiss score was 5, as the cells were pleomorphic and distributed in various round blunt cell cords, with extensive necrosis, and irregular karyotype, and capsular invasion (13).

Differential diagnosis of ACC and sex cord-stromal tumor, compared with ACC, first, the morphologic of sex cord-stromal tumor cells was not arranged in varying round blunt cell cords and cord-like structures, but were large, multi-shaped and densely clumped, and the cytoplasm was acidophilic or lipoid vacuolar with hematoxylin and eosin staining. Second, although the immunophenotype of sex cord-stromal tumor are also positive for inhibin and melan A, the other markers are negative for Syn, CK, CgA. The diagnosis sex cord-stromal tumor is excluded (21, 22).

Abdominal CT showed no adrenal mass in either adrenal gland, ruling out an adrenal origin. Combined with the clinical findings, tumor cell morphology, immunohistochemical results, and Weiss score, the final pathologic diagnosis was primary nonfunctional ectopic ACC of the lung. Adjuvant chemotherapy was provided for four cycles, and the patient had a DFS of 23 months.

Ectopic ACC in the lungs has not been previously reported. Representative ectopic adrenal tissues and neoplasms are located in anatomical organs, such as the urethral tract (23), kidney (9), ovary (10), abdominal wall (6), liver (20), retroperitoneum (11, 12), gastric wall (19), spinal canal (8), thorax (24), and lung (25). Although ectopic adrenocortical tissues and neoplasms may also be present at any anatomical location, they rarely originate in the lungs. PubMed search yielded only three reports of ectopic adrenal tissues located in the lung. First, Wadhwani et al. reported a case of ectopic adrenal tissue in the paratracheal region incidentally found during the autopsy of a 99-year-old woman. The tissue was composed of both cortical and medullary cells (24). Second, Armin et al. reported a case of congenital cytomegalic adrenal tissues in the lung of a 5-day-old infant found on autopsy (25). Third, Bozic et al. reported a case of ectopic adrenal cortex and cytomegaly in the lung found on the autopsy of a newborn (26). **Table 1** summarizes the main features and follow-up results of representative reported cases.

**Table 1:** Ectopic adrenocortical tissues, adenomas and carcinomas of representative reported cases.

Case No	Authors	Age/Gender	Diameter	Location	Functional or nonfunctional	Radiological aspect	Findings of autopsy or Surgery	Pathological diagnosis	Treatment	Follow up
1	Current case	71Y/M	6.7cm	lung	nonfunctional	5.3×5.8×6.0cm	tan-brown to tan-yellow MASS NA	ectopic ACC	tumor resection	DF 22 months
2	Shigematsu et al (23)	99Y/ F	0.5 cm	paratracheal region	functional	ND	gray-brown nodule about 5 mm NA	ectopic adrenal tissues compatible with adrenal cortex and medulla.	ND	ND
3	Armin et al (24)	5D/M	ND (two nodules)	lung	functional	ND	nodule ND	ectopic cytomegalic adrenal tissues	ND	ND
4	Bozic et al (25)	2D/F	ND (one nodules)	lung	ND	ND	ND	ectopic adrenal cortex with cytomegaly	ND	ND
5	Anuk et al (20)	1-16Y/M (Total 15) +30Y/M+70Y/F	mean 0.25 cm (range 0.2 cm -0.5 cm).	hernia sac, spermatic cord, testis, uterus	nonfunctional	ND	Yellow orange color tissues NA	ectopic adrenal cortical tissues	tumor resection	ND
6	Liu et al (8)	27Y/F	2.7 cm	renal hilum	nonfunctional	CT: a well-circumscribed, round, soft-tissue mass	ND, NA	ectopic adrenocortical adenoma	tumor resection	DF for 3 months
7	Chentli et al (7)	34Y/F	14 cm	right ovary	functional	CT: 14 cm right ovarian mass	a brown lobulated mass measuring 14.5×13.7cm	ectopic ACC	tumor resection	died on the second post-surgical day
8	Chen et al (9)	44Y/M	6cm	liver	nonfunctional	CT:7.8cm enhanced mass	Solid and yellowish-grey mass with clear margin NA	Ectopic adrenocortical oncocytic adenoma	tumor resection	ND
9	Ren et al (12)	72Y/F	3cm	gastric wall	nonfunctional	CT:15 mm × 25 mm abnormal enhanced nodule	30 mm × 30 mm mass with medium density NA	ectopic adrenal cortical adenoma	tumor resection	ND
10	Zhou et al (6)	77Y/M	30 cm	in front of the posterior peritoneum	nonfunctional	CT:30 cm × 15 cm × 8 cm tumor	a 30 cm × 15 cm × 8 cm tumor NA	ectopic ACC	tumor resection	DF for 9 months
11	Wright et al (11)	68Y/M	3.5 cm	retroperitoneal	nonfunctional	CT: a 8.3×6.0×1.6 cm mass	a tan, round, mass with nodular heterogenous borders NA	ectopic ACC	tumor resection、adjuvant radiation (5000 cGy) and adjuvant mitotane therapy	DF for 24 months
12	Konstantinov et al (13)	55Y/F	25 mm	spinal canal	nonfunctional	MRI: epidural formation18×25 mm nodule	a small knot at the base of the ponytail. NA	ectopic adrenocortical adenoma	resection	ND

Y, year; D, day; M, male; F, female; ACC, adrenocortical carcinoma; ND, not described; DF, disease-free; NA, normal adrenal.

In ectopic remnants of adrenal tissues, cortical tissues are usually seen alone, and medullary tissues are seen rarely. They are usually found incidentally during surgeries or autopsies (23, 24). Ectopic adrenal tissues may develop hyperplasia, adenoma, or carcinoma. These tumors are usually benign and nonfunctional (8, 9, 19, 20) and occasionally malignant and functional (6, 10, 12). They can produce glucocorticoids, androgens, or other sex hormones and cause clinical symptoms, such as Cushing’s syndrome, or clinical signs of virilization (4, 27).

The etiology of ectopic adrenal tissues and neoplasms in the lungs and other organs is unclear. Nevertheless, two hypotheses have been proposed. Bozic et al. suggested that mesothelial cells are located in the primordial mesenchyme of the wall of the dorsal coelom adjacent to the dorsal mesentery and primitive genitourinary structure, where they differentiate into elements that form the fetal adrenal tissue. Because the peritoneum and pleura have the same mesodermal origin as the adrenal gland, the primitive pleural mesothelium, which lies between the ramifications of the bronchial tree buds, can differentiate into ectopic adrenal tissue of the lung (26). Another possibility is that true adrenal heterotopia arises as a failure to separate the developing adrenal gland from the coelomic mesothelium, thus allowing partial incorporation into the adjacent organs (28).

Ectopic adrenal tissues may develop hyperplasia, adenoma, or carcinoma, similar to normal tissues. Therefore, we speculate that ectopic ACC in the lung of this present patient was caused by dislocation or self-differentiation of mesenchymal cells in the embryonic period, which then developed into carcinoma.

In conclusion, nonfunctional ectopic ACC in the lung is an extremely rare neoplasm that can be easily misdiagnosed as primary lung cancer or lung metastasis, both preoperatively and on postoperative pathological examination. This report may provide clues to clinicians and pathologists.

## Data availability statement

The original contributions presented in the study are included in the article/supplementary material. Further inquiries can be directed to the corresponding author.

## Ethics statement

The studies involving human participants were reviewed and approved by Caoxian People’s Hospital. The patients/participants provided their written informed consent to participate in this study.Written informed consent was obtained from the individual(s) for the publication of any potentially identifiable images or data included in this article.

## Author contributions

LN collected information, wrote the manuscript and literature review; Z-FF and ZW provided figures and pathology review. AG, SW, QD, YL and YS supervised the project and reviewed the manuscript.
